# Comparison of different rhythmic auditory stimuli on prefrontal cortex cortical activation during upper limb movement in patients with Parkinson’s disease: a functional near-infrared spectroscopy study

**DOI:** 10.3389/fneur.2024.1336268

**Published:** 2024-02-27

**Authors:** Jie Wang, Yingqi Li, Yingpeng Wang, Congxiao Wang, Shuyan Qie, Zhaohui Jin, Wenjun Du

**Affiliations:** ^1^Beijing Rehabilitation Hospital, Capital Medical University, Beijing, China; ^2^Parkinson Medical Center, Beijing Rehabilitation Hospital, Capital Medical University, Beijing, China

**Keywords:** Parkinson’s disease, prefrontal lobe, rhythmic auditory stimulation (RAS), upper limb, fNIRS

## Abstract

**Background:**

A large number of literatures show that rhythmic auditory stimulation (RAS) can effectively improve Parkinson’s disease (PD) patients’ gait speed, frequency and speed. Its application and curative effect on upper limb motor function is relatively few.

**Objective:**

By studying the immediate effect of RAS with different rhythms on the prefrontal cortex (PFC) blood oxygen response during upper limb movement in PD patients, this study discusses the potential neurophysiological mechanism of RAS on upper limb movement in PD patients, which is expected to provide guidance for patients with upper limb dysfunction such as Parkinson’s disease.

**Methods:**

In this study, 31 PD patients with upper limb static tremors were recruited to complete the nail board task on the healthy upper limb under the baseline rhythm, slow rhythm and fast rhythm provided by the therapist. At the same time, fNIRS was used to observe the blood oxygen response of PFC.

**Results:**

There was no significant main effect onsidein all brain regions (*p* > 0.05), and there was no interaction between rhythm and side (*p* > 0.05); Except lPFC, the main effect of rhythm in other brain regions was significant (*p* < 0.05), and ΔHbO increased with the change of rhythm. Paired analysis showed that there were significant differences in ΔHbO between slow rhythm and baseline rhythm, between fast rhythm and baseline rhythm, and between slow rhythm and fast rhythm (*p* < 0.05); The ΔHbO of rPFC, lDLPFC and rDLPFC were significantly different between slow rhythm and fast rhythm (*p* < 0.05); there were significant differences in the ΔHbO of BA8 between slow rhythm and baseline rhythm, and between slow rhythm and fast rhythm (*p* < 0.05).

**Conclusion:**

RAS may be a useful upper limb rehabilitation strategy for PD patients with upper limb dysfunction. At the same time, RAS with different rhythms also have different responses to PFC blood oxygen during upper limb movement in PD patients, so that we can design interventions for this kind of cortical mechanism. Identifying the neurophysiological mechanism of RAS on upper limb movement in PD patients may help clinicians customize rehabilitation methods for patients according to clues, so as to highly personalize upper limb training and optimize its effect.

## 1 Introduction

Parkinson’s disease (PD) is a degenerative disease of the central nervous system, with clinical symptoms mainly characterized by movement disorders, among which tremor is one of the most common symptoms of Parkinson’s disease (1). Parkinson’s disease tremor often occurs in one side of the limb, and the onset of left or right limbs is random. Upper limb tremor is one of the symptoms that PD patients exhibit during their early onset. After its onset, it seriously affects their ability to take care of themselves in daily life (2), leading to a loss of workability and decreased quality of life. The standard methods for treating and interfering with PD tremor symptoms mainly rely on drug treatment, supplemented by surgical treatment. However, drug treatment has toxic side effects and drug resistance issues, and surgical treatment also has certain risks and medical costs. Music therapy is an emerging interdisciplinary treatment technology, which is increasingly used in clinical intervention research due to its low investment, no adverse reactions, and easy patient acceptance.

Rhythmic auditory stimulation (RAS) is a training method that enhances motor ability by providing rhythmic stimuli (music, rhythm, etc.) to the motor center. It can achieve specific functional rehabilitation goals for neuromotor disorders by widely triggering and promoting sensory, motor, and cognitive neural activity in the brain (3). A large number of literatures have shown that the application of RAS in gait can effectively improve the gait speed, gait frequency, and gait speed of PD patients (4). There are also studies indicating that appropriately faster RAS can lead to faster walking speed in PD patients (5), but there is little research on the response of upper limb movement to RAS in PD patients. A study (6) examined the effect of RAS on upper limb movement in PD patients and found that faster RAS led to faster upper limb movement in both PD patients and healthy individuals. However, the study did not elucidate the neural mechanisms underlying the impact of RAS on upper limb movement in PD patients.

The ability to synchronize body movements with external rhythm signals is almost unique to humans, and human perception of rhythm is a complex cognitive process. The main external manifestation of rhythm perception is spontaneous synchronized limb movements, while the internal manifestation in the cognitive process is the synchronized oscillation of the rhythm of internal attention and external stimuli. When a person is exposed to rhythmic stimuli, the brain will oscillate synchronously with external rhythmic signals to generate anticipatory attention, which drives us to anticipate the occurrence of time at the beat point and helps us cope with external stimuli (7). Rhythmic external stimuli can attract anticipatory attention, manifested as facilitating the processing of stimuli when they appear at rhythmic points, or improving their accuracy (8). Top-down signals from the prefrontal cortex (PFC) are crucial for cognitive control and can selectively focus on environmental inputs. Positron emission computed tomography (PET) and functional magnetic resonance imaging (fMRI) studies have found that PFC is activated in auditory tasks that require top-down attention (9). PFC has also been proven to play an important role in human auditory attention (10). The main pathological changes of PD are progressive degeneration and loss of dopaminergic neurons in the substantia nigra, degeneration of the nigrostriatal circuit, which leads to inhibition of the direct pathway in the striatum and inhibition of the indirect pathway, resulting in abnormal function of the striatum thalamus cortex circuit. The decrease in dopamine activity in the frontal lobe is an important factor leading to decreased executive function (11). The frontal striatal circuit of the patient is damaged to varying degrees, but PD patients still need compensatory activation of the frontal nerve to maintain stable movement (12). Using PET research, Thaut et al. (13) found that when healthy adults tap their fingers at different rhythms of RAS, there is a difference in the recruitment data of neurons in the frontal lobe, with faster rhythms of RAS mobilizing neurons being more significant. However, the mechanism of RAS with different rhythms in the upper limb movement of PD patients is still unclear.

Functional near-infrared spectroscopy (fNIRS) is a new type of brain functional imaging technology developed in recent years. Its principle mainly relies on the absorption of light by brain tissue to detect changes in blood oxygen levels during brain activity. Through certain image restoration and reconstruction, it can further obtain near-infrared optical images of brain activity, and then analyze functional activity information of the cerebral cortex. Compared to other non-invasive brain functional imaging technologies such as PET, fMRI, and EEG, fNIRS has the advantages of safety, noninvasive, easy to move, anti-motion interference, anti-electromagnetic interference, high spatiotemporal resolution, and allowing long-term monitoring. Due to its advantages, fNIRS has been widely used in clinical monitoring and cognitive science research in neurology, neonatology, and rehabilitation (14, 15).

This study intervened in the upper limb nail board task of PD patients through auditory stimulation schemes with different rhythms, and evaluated the activation of PFC in the task state using fNIRS. The study explored the brain mechanism effects of PD patients on auditory stimuli with different rhythms, and judged whether there were differences in attention, cognitive control, and stimulus recognition.

## 2 Materials and methods

### 2.1 Participants

This study recruited 31 PD patients. This study was approved by the Hospital Ethics Committee (Ethics Number: 2022bkky-088).

#### 2.1.1 Inclusion criteria

- All participants were diagnosed with primary Parkinson’s disease according to the clinical diagnostic criteria of Parkinson’s disease in the brain bank of the British Parkinson’s Disease society.
- Hoehn and Yahr (H & Y) Parkinson’s scale score is 2–3.
- In UPDRS III, the score of limb static tremor was at least 1.
- The age is 30–70 years old.
- Right-handed; dominant side, right.
- Stable condition.
- Serious verbal and cognitive impairment, no command.
- All study participants signed written informed consent.

#### 2.1.2 Exclusion criteria

- Various secondary Parkinson’s syndrome or Parkinson’s superposition syndrome.
- Have definite central nervous system diseases or brain surgery in the past.
- Accompanied by musculoskeletal or peripheral nerve diseases.
- Previous history of drug or alcohol abuse.
- Patients with visual or visual impairment.
- Those who are participating in other clinical trials that affect the evaluation of the results of this study.
- Patients who cannot sign informed consent.

The general characteristics of all participants are shown in Table 1.

**Table 1:** General characteristics of participants.

	RAS group
Gender (male/female)	13/18
Affected side (L/R)	15/16
Age (years)	58.2 ± 8.4
Height (cm)	163.94 ± 7.11
Weight (kg)	61.63 ± 8.87
Course of disease (years)	6.68 ± 4.02
H & Y (2/2.5/3)	14/9/8
UPDRS III limb static tremor score	1.13 ± 0.34

### 2.2 Experimental task

Before the test, understand the performance level of each participant and measure the movement speed without RAS assistance (baseline speed) (4, 6). In the actual test, the participants completed the nail board task on the healthy upper limb under the RAS (baseline rhythm) equal to 100% of the baseline speed, RAS (slow rhythm) equal to 50% of the baseline speed and RAS (fast rhythm) equal to 150% of the baseline speed provided by the therapist.

### 2.3 fNIRS

#### 2.3.1 fNIRS data acquisition

##### 2.3.1.1 Test instrument

The NIRS data were measured by ETG-4000 near infrared brain function imager produced by Hitachi in Japan. During quiet and specified exercise, the relative variables of brain frontal lobe oxygenated hemoglobin (HbO), deoxyhemoglobin (HbR) and total hemoglobin concentrations (sampling frequency = 10 Hz, number of measurement channels = 22, source detector distance = 3 cm).

##### 2.3.1.2 Test environment

The test was conducted in a quiet room, and only the tester and subjects were present at each test; Before the test, the subjects were subjected to the experimental task for half an hour. After the training, they began to rest for 5 min; the subject took the end seat, relaxed as much as possible and kept the head still.

##### 2.3.1.3 Test position

**Figure 1:**
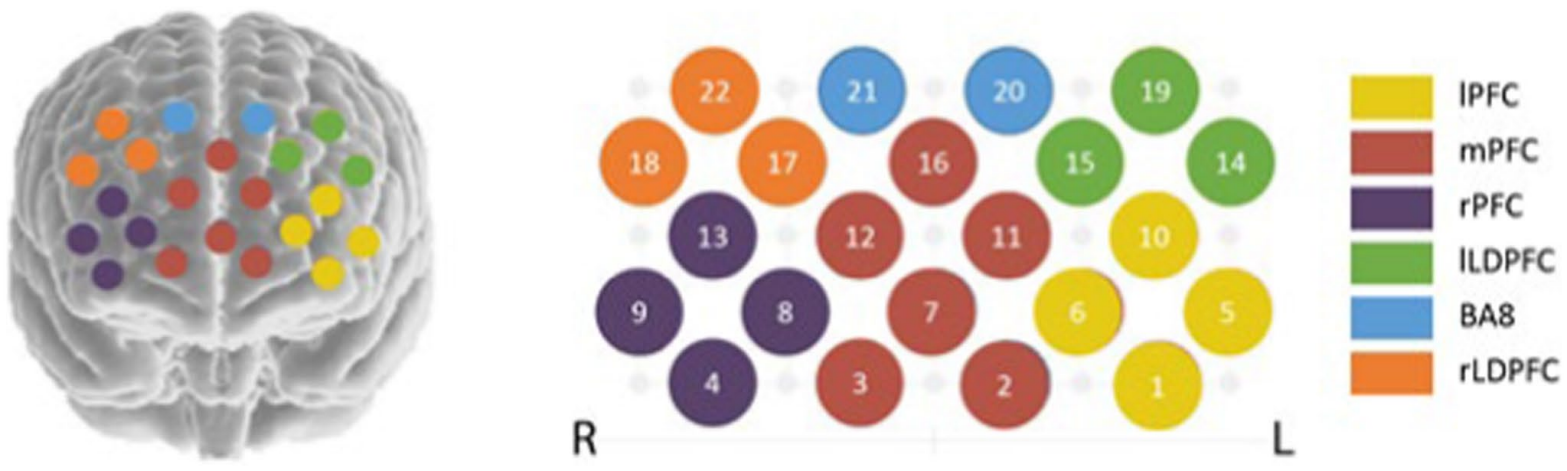
Schematic diagram of channel setting. lPFC (CH1, CH5, CH6, CH10); mPFC (CH2, CH3, CH7, CH11, CH12, CH16); rPFC (CH4, CH8, CH9, CH13); lLDPFC (CH14, CH15, CH19); BA8 (CH20, CH21); rLDPFC (CH17, CH18, CH22).

Select 3 × 5. In order to determine the location of the region corresponding to each channel, the 10–20 system diagram used to locate the functional region of the brain in EEG is used. Fix the head cover with the probe on the forehead of the brain, debug the probe, and start the formal test after all the display signals of the channel are connected. The specific channel layout is shown in Figure 1 (16).

#### 2.3.2 fNIRS data preprocessing

NIRS-SPM software package is used for data preprocessing. Manually mark the original data with obvious motion artifacts or damaged parts of the channel, and detect and correct the artifacts. Apply 0.01–0.14 Hz band-pass filtering to eliminate interference caused by heartbeat (0.8–2.0 Hz), respiration (0.1–0.33 Hz) and Meyer wave (0.1 Hz or lower). Then, according to the modified Beer Lambert law, the HbO of each channel is calculated with the filtered data. In addition, motion artifacts are removed based on moving standard deviation and spline difference (17–19).

### 2.4 Statistical analysis

The effect of rhythmic auditory stimulation at different speeds on the prefrontal cortex during upper limb movement in PD patients was defined as ΔHbO (i.e., ΔHbO = average HbO during upper limb movement − average HbO at rest). SPSS 22.0 was used for statistical processing and analysis. Two-way repeated measures ANOVA was used to determine the effect of side and speed on concentration. Both side and speed were within subject factors. Conduct a Mauchly’s test of sphericity on the data. If *p* < 0.05 and dissatisfied with the football hypothesis, use the Greenhouse & Geisser method for correction. If the interaction between side and speed is significant (*p* < 0.05), analyze the individual effects of each factor; If the interaction was not significant (*p* < 0.05), the main effects of each factor were analyzed. The post pairwise comparison was corrected by Bonferroni method.

## 3 Result

There was no significant main effect onsidein all brain regions (*p* > 0.05), and there was no interaction between rhythm and side (*p* > 0.05); except LPFC, the main effect of rhythm in other brain regions was significant (*p* < 0.05), ΔHbO increases with the change of rhythm. See Tables 2, 3.

**Table 2:** Effects of RAS with different rhythms on ΔHbO of PFC during upper limb movement in PD patients (mol/L).

		Slow rhythm	Baseline rhythm	Fast rhythm
lPFC	Uninvolved side	0.021 ± 0.035	0.032 ± 0.042	0.035 ± 0.084
	Involved side	0.027 ± 0.049	0.031 ± 0.038	0.045 ± 0.053
mPFC	Uninvolved side	0.009 ± 0.036	0.030 ± 0.046	0.040 ± 0.055
	Involved side	0.018 ± 0.048	0.021 ± 0.032	0.042 ± 0.049
rPFC	Uninvolved side	0.019 ± 0.026	0.040 ± 0.050	0.045 ± 0.064
	Involved side	0.024 ± 0.043	0.030 ± 0.032	0.045 ± 0.024
lDLPFC	Uninvolved side	0.017 ± 0.028	0.030 ± 0.037	0.040 ± 0.050
	Involved side	0.029 ± 0.037	0.028 ± 0.032	0.047 ± 0.052
rDLPFC	Uninvolved side	0.014 ± 0.026	0.030 ± 0.036	0.034 ± 0.071
	Involved side	0.021 ± 0.046	0.028 ± 0.022	0.046 ± 0.052
BA8	Uninvolved side	0.006 ± 0.022	0.021 ± 0.035	0.037 ± 0.050
	Involved side	0.011 ± 0.048	0.021 ± 0.022	0.038 ± 0.059

**Table 3:** Results of repeated measurement analysis of variance of two factors: sides and different rhythm.

	Lateral classification		Rhythm		Lateral * rhythm	
	*F*-value	*p*-value	*F*-value	*p*-value	*F*-value	*p*-value
lPFC	0.626	0.435	2.001	0.144	0.202	0.756
mPFC	0.012	0.913	14.309	<0001	1.051	0.344
rPFC	0.070	0.793	7.483	0.001	0.653	0.494
lDLPFC	0.835	0.368	7.636	0.003	0.781	0.463
rDLPFC	0.883	0.355	7.626	0.001	0.501	0.609
BA8	0.116	0.736	10.018	0.001	0.129	0.879

**Figure 2:**
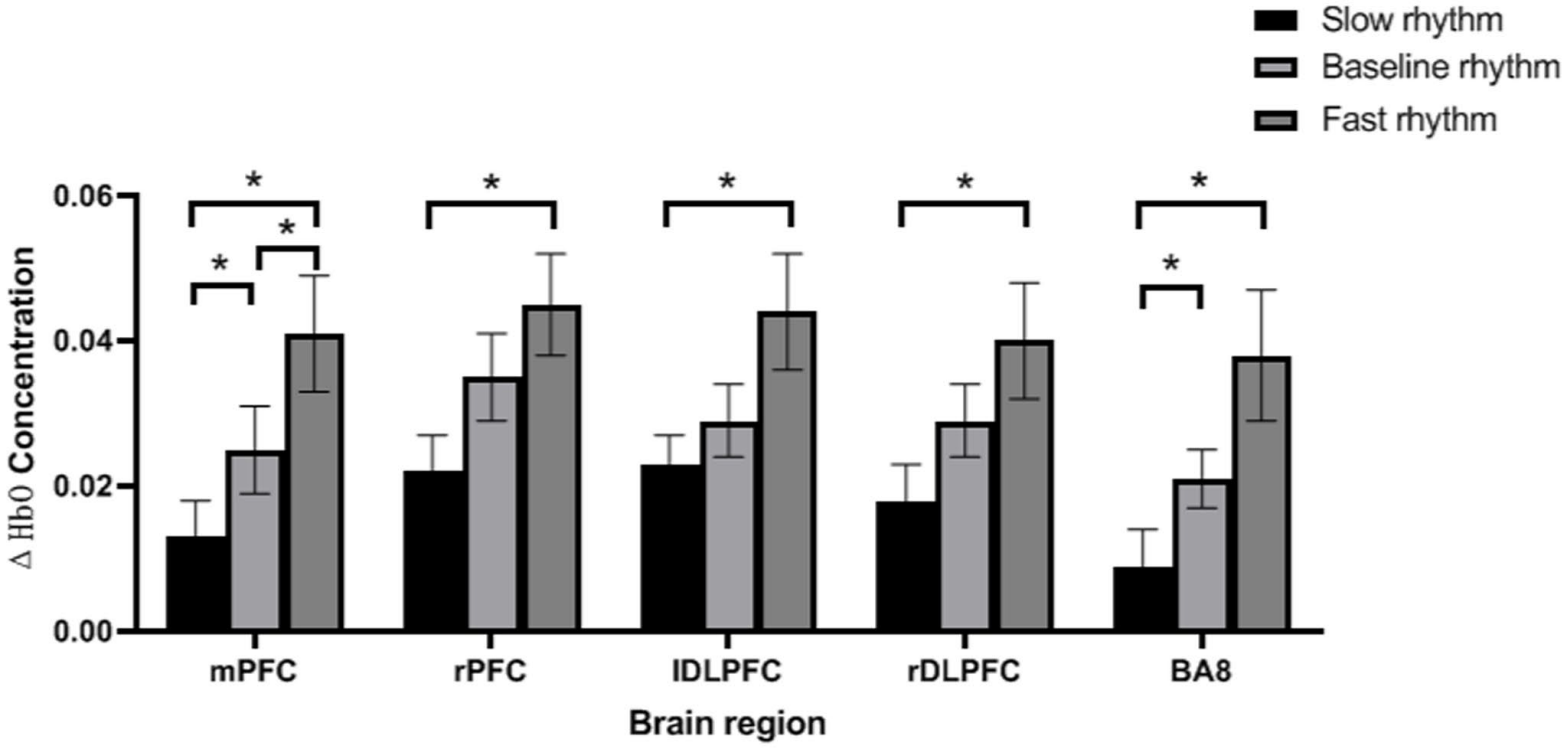
Paired analysis results. ^*^p < 0.05, error bar represents the standard deviation of the mean.

Paired analysis showed that there were significant differences in ΔHbO between slow rhythm and baseline rhythm, between fast rhythm and baseline rhythm, and between slow rhythm and fast rhythm (*p* < 0.05); ΔHbO of rPFC, lDLPFC and rDLPFC were significantly different between slow rhythm and fast rhythm (*p* < 0.05); there were significant differences in the ΔHbO of BA8 between slow rhythm and baseline rhythm, and between slow rhythm and fast rhythm (*p* < 0.05). See Figure 2.

## 4 Discussion

Some studies suggest that there are metronomes in the human body that can guide the body to produce rhythmic movements such as upper limb waving and shaking (20). RAS involves external auditory stimulation, during which the internal metronome of the body tends to align with the external rhythm, allowing muscles to move in a natural way, thereby improving the effectiveness of various training contents (21). External rhythmic cues may improve motor dysfunction in patients by promoting synchronization of brain neural networks or strengthening attention control. One of the main mechanisms of RAS may be based on training for attention control and inhibition (22). In order to maintain alignment with the target, the subject is required to focus like a laser on the “rhythm and tone feedback” of the target (for a period of time) and turn off or suppress attention interference generated by internal or external stimuli (23). The prefrontal cortex extracts information related to the regularity of cognitive experience, thereby regulating thoughts and behaviors, playing a role in the cognitive neural foundation of the brain.

This study found that in PD patients, faster RAS leads to a faster increase in HbO in the frontal lobe during upper limb movement, and no interaction between lPFC and RAS was found. In addition, when patients hear each rhythm of RAS, there is no difference in the impact of involved and uninvolved upper limb movements on PFC. Finally, mPFC is most sensitive to the impact between each rhythm, while the remaining brain regions only exhibit differences between slow and fast rhythms.

We found that in PD patients, faster RAS leads to a more pronounced HbO response in the frontal lobe, which is consistent with early studies indicating that fast-paced auditory stimuli have an impact on PFC during walking in PD patients (24). It is also consistent with the findings of Fan et al. (6) that faster RAS effectively induces faster upper limb movements. Aron et al. (25) believes that the frontal region suppresses tasks through the subthalamic nucleus (STN) network, and that voluntary inhibition of manual movement in humans depends on the right frontal basal ganglia thalamic pathway. In this study, performing nail board tasks according to different rhythms was a manifestation of inhibitory planning ability, thus the results showed that rPFC was activated while lPFC had no effect on RAS. The classic view holds that executive function is achieved through a functional network loop composed of the frontal lobe and related brain regions, especially DLPFC, which is believed to be closely involved in executive function. Previous studies have also shown that DLPFC is mainly related to executive control, decision-making, attention control, working memory processing, and reactive selection. In this study, the nailboard task not only involves aiming at the target object, but also controlling the sense of rhythm, which increases the difficulty of this action. It requires the subjects to make careful decisions, concentrate their attention, and internal timing, and reflect on the processing, rapid decision-making, and accurate execution of working memory within a limited time control range. The activation of these brain regions indicates that the subjects are correctly conducting the experiment according to the experimental requirements, this result is also consistent with previous research findings (26–28). Slow rhythm has lower requirements for the executive function of PFC, with lower activation levels in its brain regions, while fast rhythm requires more executive ability to effectively complete set goals. Its brain regions have higher activation levels, so there are significant differences between slow and fast rhythms in each brain region. Based on previous research, it is known that (29) mPFC is mainly responsible for the conversion between complex tasks and increasing anti-interference ability. In this study, changes in rhythm can enhance the interference of upper limb movements in PD patients, which may require the activation of mPFC to resist interference. Therefore, mPFC is more sensitive to changes in rhythm. However, this study found that when patients heard each rhythm of RAS, there was no interaction between the involved and non-involved upper limb movements on PFC, which may be related to the lower degree of stationary tremors in the involved limb of the subjects.

Although our research findings are meaningful, this study still has limitations and deserves further discussion. Firstly, our sample size is small, and we included mild to moderate PD patients with mild tremor symptoms. We suggest that in the future, we can study the impact of RAS with different rhythms on different degrees of tremor. Secondly, considering the number of channels available for fNIRS instruments, we only studied PFC and did not study the motor cortex. Future research should focus on the influence of whole brain mechanisms or functional connectivity between brain regions. Thirdly, we only studied the immediate impact of RAS and did not investigate the effectiveness of the training plan. Future research should develop training involving RAS and examine its impact on motor function and changes in brain regions in PD patients. Fourthly, considering the trade-off between speed and accuracy (30), faster rhythmic auditory stimuli may lead to PD patients completing nail board tasks faster but less accurately, and we did not evaluate the accuracy or variability of their movements. In the future, we need to check the accuracy of its movement. Finally, we acknowledge that our research findings are not generalizable as we only studied PD patients with right-handed hands to avoid the impact of lateralization. Suggest future research to further explore the impact of RAS on upper limb movement in PD patients through more detailed grouping.

## 5 Conclusion

In conclusion, our results show that RAS may be a useful upper limb rehabilitation strategy for PD patients with upper limb dysfunction. At the same time, different rhythmic auditory stimuli also have different responses to PFC blood oxygen during upper limb movement in PD patients, enabling us to design interventions for such cortical mechanisms. Identifying the neurophysiological mechanism of RAS on upper limb movement in PD patients may help clinicians customize rehabilitation methods for patients according to clues, so as to highly personalize upper limb training and optimize its effect.

## Data availability statement

The original contributions presented in the study are included in the article/supplementary material, further inquiries can be directed to the corresponding author.

## Ethics statement

The studies involving humans were approved by Beijing Rehabilitation Hospital Ethics Committee (Ethics Number: 2022bkky-088). The studies were conducted in accordance with the local legislation and institutional requirements. The participants provided their written informed consent to participate in this study.

## Author contributions

JW: Data curation, Investigation, Methodology, Writing – original draft. YL: Formal analysis, Writing – review & editing. YW: Methodology, Software, Writing – review & editing. CW: Resources, Writing – review & editing. SQ: Conceptualization, Supervision, Writing – review & editing. ZJ: Formal analysis, Writing – review & editing. WD: Formal analysis, Writing – review & editing.
